# Technology-assisted rehabilitation following total knee or hip replacement for people with osteoarthritis: a systematic review and meta-analysis

**DOI:** 10.1186/s12891-019-2900-x

**Published:** 2019-11-03

**Authors:** Xia Wang, David J. Hunter, Giovana Vesentini, Daniel Pozzobon, Manuela L. Ferreira

**Affiliations:** 1 Level 10 Kolling Institute, Institute of Bone and Joint Research, Royal North Shore Hospital, University of Sydney, Reserve Road, St. Leonards, Sydney, NSW 2065 Australia; 2 Department of Gynaecology and Obstetrics, Botucatu Medical School, San Paulo State University, São Paulo, Brazil

**Keywords:** Joint arthroplasty, Healthcare delivery, Telerehabilitation, Digital health, Virtual reality

## Abstract

**Background:**

To evaluate the effectiveness and safety of technology-assisted rehabilitation following total hip/knee replacement (THR/TKR).

**Methods:**

Six electronic databases were searched without language or time restrictions for relevant studies: MEDLINE, EMBASE, Cochrane Library, CINAHL, SPORTDiscus, Physiotherapy Evidence Database (PEDro); from inception to November 7th, 2018. Two reviewers independently applied inclusion criteria to select eligible randomised controlled trials (RCTs) that investigated the effectiveness of technology-based interventions, compared with usual care or no intervention for people undergoing THR/TKR. Two reviewers independently extracted trial details (e.g. patients’ profile, intervention, outcomes, attrition and adverse events). Study methodological quality was assessed using the PEDro scale. Quality of evidence was critically appraised using the Grading of Recommendations, Assessment, Development and Evaluation approach.

**Results:**

We identified 21 eligible studies assessing telerehabilitation, game- or web-based therapy. There were 17 studies (*N* = 2188) in post-TKR rehabilitation and 4 studies (*N* = 783) in post-THR rehabilitation. Compared to usual care, technology-based intervention was more effective in reducing pain (mean difference (MD): − 0.25; 95% confidence interval (CI): − 0.48, − 0.02; moderate evidence) and improving function measured with the timed up-and-go test (MD: -7.03; 95% CI: − 11.18, − 2.88) in people undergoing TKR. No between-group differences were observed in rates of hospital readmissions or treatment-related adverse events (AEs) in those studies.

**Conclusion:**

There is moderate-quality of evidence showed technology-assisted rehabilitation, in particular, telerehabilitation, results in a statistically significant improvement in pain; and low-quality of evidence for the improvement in functional mobility in people undergoing TKR. The effects were however too small to be clinically significant. For THR, there is very limited low-quality evidence shows no significant effects.

## Background

Knee or hip osteoarthritis are dominant sources of disability, affecting approximately 776 million people globally [1]. These conditions are leading contributors to the rapid increase in orthopaedic surgeries worldwide over the last decades, with most of the increase occurring in total knee (TKR) and hip replacement (THR) [2]. Given the large and increasing financial burden of these procedures, potential efficiencies in the model of care for arthroplasty patients are a matter of considerable policy interest [3]. Rehabilitation services form a core component of the care pathway for THA and TKA patients, as a means of facilitating the recovery of functional independence after surgery. Due to the increased life expectancy and the limited resources devoted to public health, the demand for effective and sustainable rehabilitation services seems mandatory in order to cope with the needs of the aging population [4].

Recently, innovative technologies have brought affordability and convenience to the healthcare consumers, such as eHealth, telemedicine, wearables, virtual reality (VR) and online educational tools [5]. A growing body of literature supports the use of telerehabilitation in improving patient satisfaction and health outcomes for a diverse range of clinical conditions, such as neurological diseases [6, 7], stroke [8], cancer [9], cardiac and pulmonary rehabilitation [10]. Compared to face-to-face rehabilitation, services delivered remotely via telephone or internet are more affordable and accessible, particularly for people living in rural areas [11]. In addition, telerehabilitation systems integrated with biosensors, accelerometers and educational software provide individualised support for people to monitor the progress of their physical rehabilitation at home, whilst allowing the therapist to intervene timely and effectively [12]. Several studies have shown that game-based or VR-assisted rehabilitation provides a motivating environment for achieving different therapeutic goals [13]. Importantly, these innovative technologies empower consumers to take an active role in decision-making and disease management, resulting in improvements of overall health awareness, adherence to treatment and satisfaction [14].

Despite the increasing popularity of available innovative health products in the market, there is insufficient evidence of their effectiveness or safety in musculoskeletal (MSK) rehabilitation. A few systematic reviews of telerehabilitation have been conducted but only yielded a handful of trials [15–17]. However, along with the rapid progress in the technologies and the growing service demand, the number of publications in this topic also increased since then, thus, it is necessary to update the evidence at a timely manner. In addition, other blooming technologies, such as game therapy and virtual biofeedback have not been well investigated. Thus, this review aimed to update the current evidence and evaluate the effectiveness and safety of technology-based rehabilitation in comparison with usual care in people undergoing TKR and THR.

## Methods

A protocol for this review was registered a priori in PROSPERO (CRD42017078924) and preliminary results were presented in a conference [18]. This systematic review with meta-analyses reported according to the Preferred Reporting Items for Systematic Reviews and Meta-Analysis (PRISMA) statement [19]. All the screening, data extraction and quality assessment were performed by two authors (XW, GV) independently and any disagreement was resolved by consensus with a third reviewer (MLF).

### Literature search

Six electronic databases were searched without language or time restrictions for relevant studies: MEDLINE, EMBASE, Cochrane Library, CINAHL, SPORTDiscus, Physiotherapy Evidence Database (PEDro); from inception to November 7th, 2018. The search strategy was developed by a research librarian and contained both controlled vocabulary and free text terms (Additional file 1: Appendix 1). The initial search strategies included lumbar spinal surgeries, as lumbar spinal surgeries are also highly prevalent in orthopaedic surgeries. However, there is only one study in lumbar spinal surgeries has been identified, so we only reported results for TKR and THR in this paper.

### Study selection

The population of interest was people undergoing rehabilitation after elective TKR and THR. Eligible studies were randomised controlled trials (RCTs) that investigated the effectiveness of any technology-based intervention, in isolation or in combination with other interventions, compared with usual care and no treatment. Technology-based interventions were defined as any type of health-related services such as education, monitoring or treatment delivering via telecommunication technologies, internet, software or VR devices. The primary outcomes were pain and function. The secondary outcomes were quality of life, adherence, user experience and safety.

### Data extraction

Trial details, including patients’ clinical profile, intervention, outcomes, attrition and adverse events (AEs), were recorded on a dedicated trial description form. Outcome data included mean score, mean difference (MD) between groups, odds ratios (ORs), risk ratios (RRs), standard deviations (SDs) and standard errors (SEs). Outcome data were extracted for short-term (immediate effect post-intervention to ≤3 months follow up), medium-term (3 to 6 months follow up) and long-term (≥ 6 months follow up) assessments. When more than one follow-ups were performed within each category, data from the shortest period of follow up were extracted.

### Study methodological quality

The PEDro scale [20] was used to determine the methodological quality of each study. This 10-point scale is a valid assessment tool for the internal and external validity of randomised clinical trials, with acceptable reliability: intraclass correlation coefficient (ICCs) for inter-rater reliability of 0.56 for the total score; and 0.68 for consensus ratings [21, 22]. When available, quality scores were extracted from the PEDro database (www.pedro.org.au). Studies with a score of 7 or greater were considered “high quality” [23].

### Quality of evidence

The Grading of Recommendations, Assessment, Development and Evaluation (GRADE) approach was used to appraise the quality of evidence for making clinical practice recommendations [24]. The quality of evidence was initially considered as high and downgraded based on five criteria: high risk of bias (e.g. > 25% of participants for studies with a PEDro score of ≤6), inconsistency of results (I^2^ > 50%), indirectness (comparison of different populations and interventions), imprecision (e.g. sample size < 400, 95% CI overlaps no effect) and publication bias (visual inspection of funnel plots and Egger’s regression test) [24].

### Data synthesis and analysis

For the meta-analyses, whenever possible, outcomes were converted to a standard scale. For all variables with the same outcome, MDs or standardised MDs (SMD) with a 95% CI were calculated. Trials deemed clinically homogeneous were grouped according to 1) outcome measure, 2) follow-up duration and 3) surgery type. Between-trial heterogeneity was evaluated by visual inspection of the forest plots [25] and the I^2^ statistic (I^2^ < 50%: low to moderate; I^2^ ≥ 50%: substantial; I^2^ > 75% considerable heterogeneity) [26]. Random effect models were used to pool study results with considerable heterogeneity (i.e. I^2^ > 75%) [26]. Meta-analyses were performed using Review Manager, Version 5.3.

## Results

### Results of the search

**Fig. 1:**
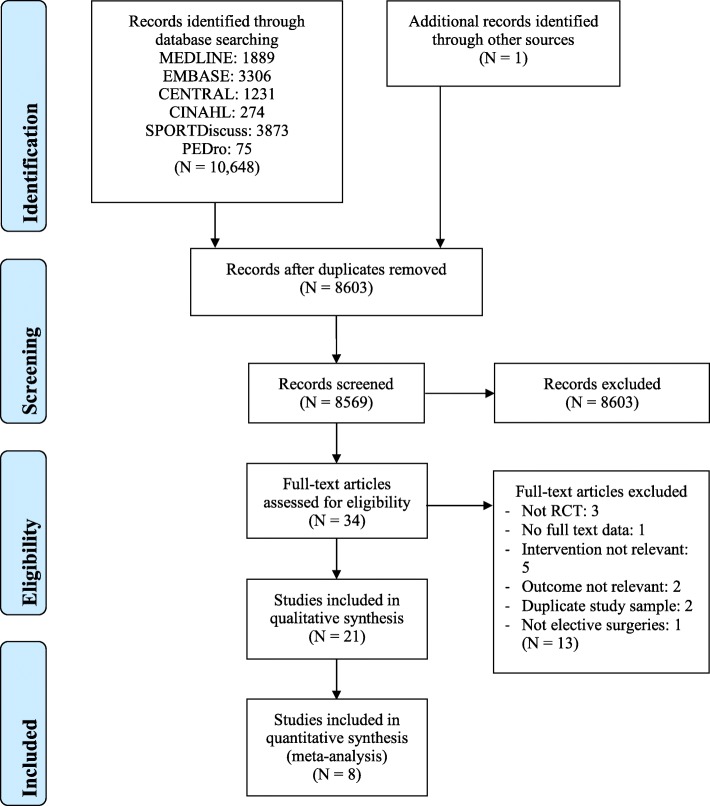
PRISMA flowchart

In total, 21 RCTs (from 20 publications, *N* = 2971, mean age = 65.2 years old) were included after the screening of 8603 relevant studies retrieved from various databases. Figure 1 shows the PRISMA flowchart for the screening. The characteristics of included participants, interventions, outcomes and main findings are detailed in Table 1.

**Table 1:** Characteristics of the included studies according to surgery and intervention types

Study	Sample size	Age (y)*	Female	Condition	Intervention	Control	Length of intervention	Outcomes	Time points	Results	PEDro scores
Total knee replacement											
Telephone-based rehabilitation											
Chen et al. 2016 (China)	Total: 202 IG: 101 CG: 101	66.6	68.1%	Knee OA	Standardised rehabilitation programme monitored via telephone support and counselling	Standardised in-patient rehabilitation programme	3 calls (5–10 min each) at week, 1, 3 and 6	VAS pain; ROM; SF-36; Beck Depression Inventory scale	Post-surgery baseline, 3, 6, 12 months	The mean exercise time and total days in the IG group were significantly higher than CG (P < 0.01). The pain and Beck Depression Scale scores of the IG were significantly lower than those of the CG (*P* < 0.01) 3 months after TKA. The IG had greater improvement on MCS scores and active ROM (P < 0.01) after TKA.	7/10
Han et al. 2015 (Australia)	Total: 390 IG: 194 CG: 196	64.8	53.0%	Knee OA	Home exercise programme monitored via telephone support and counselling	Usual care includes access to clinic-based outpatient physiotherapy after discharge	1 call/week for 6 weeks	WOMAC; ROM; 50-ft walk time; adverse events; hospital readmission	Post-surgery baseline, 6 weeks	No significant differences between groups were observed, respectively, for WOMAC pain (MD: 0.1; 95% CI: − 0.7, 0.9), physical function (MD: 0.04; 95% CI: − 2.5, 2.6), knee flexion (MD: − 1.1; 95% CI: − 4.1 to 1.9), knee extension (MD: 0.2; 95% CI: − 1.6 to 1.2), or the 50-ft walk time (MD: − 0.04; 95% CI: − 0.8, 0.7) at 6 weeks after surgery. No statistically significant difference between groups was observed in the number of hospital readmissions.	8/10
Kramer et al. 2003 (Canada)	Total: 160 IG: 80 CG: 80	68.4	59.0%	Knee OA	Home exercise monitored via telephone support and counselling	Common home exercise + out-patient clinic-based rehabilitation	At least 2 calls (10~30 min each) between week 2–6 and 7–12	WOMAC; 6MWT; ROM; SF-36; Knee Society Clinical Rating scale; 30-s stair test	Post-surgery baseline, 6 weeks, 3, 6, 12 months	No statistically significant differences between groups were observed for the pain outcome measures (WOMAC pain scores and Knee Society Clinical Rating scale) and mobility (30-s stair test and 6MWT) at 12- or 52-weeks post-surgery.	6/10
Park et al. 2017 (South Korea)	Total: 40 IG: 21 CG: 19	50–60 years: N = 18; 70–80: *N* = 22	89.5%	Knee OA	Telephone support and counselling only	SMS texts after discharge	6 calls at week 1, 3, 5, 7, 9 and 11	WOMAC global; Korean-style ADL; life satisfaction index-Z	Pre-surgery baseline, 1, 3 months	No statistically significant differences between groups were observed for WOMAC, ADL, and life satisfaction.	5/10
Szöts et al. 2016 (Demark)	Total: 117 IG: 59 CG: 58	67.6	66.7%	Knee OA	Conventional rehabilitation programme monitored via telephone support and counselling	Conventional in-patient and out-patient treatment of TKA	2 calls (11–48 min each) at day 4 and 14	WOMAC; SF-36; general self-efficacy scale	Post-surgery baseline, 1, 3 months	No statistically significant differences between groups were observed on all WOMAC scores. However, significant differences in scores were identified in favour of the IG on general self-efficacy (between-group difference: 2.0; 95% CI: 0, 3.0) and physical function scale of SF-36 (between-group difference: 10.0; 95% CI: 0, 20.0) at 1 month after TKA, but this effect was not seen at 3 months.	8/10
Video-teleconferencing											
Moffet et al. 2015 (Canada)	Total: 205 IG: 104 CG: 101	66.0	45.0%	Knee OA	Standardised rehabilitation programme via in-home videoconferencing	Standardised rehabilitation programme via face-to-face home visits	16 sessions (45–60 min each) over 2 months	WOMAC; 6MWT; ROM; KOOS; timed stair test	Pre-surgery baseline, 2, 4 months	Non-inferiority of the IG compared with CG for all WOMAC scores, 6MWT, KOOS scores, ROM and timed stair tests at 2 months or 4 months after hospital discharge.	8/10
Tousignant et al. 2011 (Canada)	Total: 41 IG: 21 CG: 20	66.0	NR	NR	Functional rehabilitation via videoconferencing	Usual home care services referred by the institute	2 sessions/week (60 min each) for 8 weeks	WOMAC; ROM; TUGT; SF-36; Berg balance scale; 30s chair-stand test; Tinetti test; Functional Autonomy Measurement System	Post-surgery baseline, end of treatment, 2 months	No statistically significant differences between groups were observed for all clinical variables. The CG had greater improvement on WOMAC difficulty (climbing stairs, walking) (*P* = 0.047), physical functioning (*P* = 0.019) and less bodily pain (*P* = 0.014) after 2 months.	5/10
Russell et al. 2011 (Australia)	Total: 65 IG: 31 CG: 34	67.9	41.0%	NR	Standard rehabilitation programme via internet-based videoconferencing + motion analysis tools	Standard out-patient clinical rehabilitation	1 session/week (45 min each) for 6 weeks	VAS pain; WOMAC; TUGT; ROM; Patient-Specific Functional Scale; quadriceps lag; limb girth knee; limb girth calf; Gait Assessment Rating Scale, compliance and satisfaction	Post-surgery baseline, 6 weeks	No statistically significant differences between groups were observed for knee flexion and extension, muscle strength, limb girth, pain, TUGT, QoL, and clinical gait and WOMAC scores at 6 weeks after intervention. Better outcomes were found in the IG for the Patient-Specific Functional Scale (between-group difference: −1.08; 95% CI: − 1.86, − 0.30) and the WOMAC stiffness (between-group difference: 1.46; 95% CI: 0.24, 2.68) at 6 weeks. The intervention was well received by participants, who reported a high level of satisfaction with this novel technology.	8/10
Piqueras et al. 2013 (Spain)	Total: 142 IG: 72 CG: 70	73.3 ± 6.5	83.0%	Knee OA	Weight-bearing functional exercise via a videoconference software with a 3D avatar + wireless sensors (accelerometer and gyroscopes) + web portal for therapist to evaluate patient data	Standardised rehabilitation programme	1 session/day (60 min each) for 10 days (supervised sessions for 5 days followed by home self-care sessions for 5 days)	VAS pain; WOMAC; TUGT; ROM; quadriceps muscle strength; hamstring muscle strength	Post-surgery baseline, 2 weeks; 3 months	Active extension ROM had a greater increase at 5 days post-surgery (*P* = 0.045), but the increase became equal at 3 months. IG achieved a greater increase in muscle strength (*P* = 0.011) and it was maintained after 3 months (*P* = 0.018). CG had a lower baseline level for TUGT, therefore had a greater increase at 3 months (*P* = 0.008).	6/10
Game-based therapy/Visual biofeedback											
Christiansen et al. 2015 (U.S.)	Total: 26 IG: 13 CG: 13	67.4	46.2%	Knee OA	In-patient post-operative physical therapy + home exercise programme + weight-bearing biofeedback training with a Nintendo Wii Fit balance board	In-patient post-operative physical therapy + home exercise program	IG: 1 session/day for 6 weeks CG: 2 sessions/day for 6 weeks	Weight-Bearing Ratio; hip, knee and ankle moment	Post-surgery baseline, 6 weeks, 26 weeks	No statistically significant differences were found between groups for weight-bearing ratios, knee extension moment. FTSST improved in the IG compared with the CG at 6 (between-group difference: −2.3; 95% CI: − 4.2, − 0.4) and 26 weeks (between-group difference: − 1.3; 95% CI: − 2.3, − 0.2).	7/10
Ficklscherer et al. 2016 (Germany)	Total: 30 IG: 17 CG: 13	53.0	38.5%	TKR and ACL	Standard physiotherapy + exercise training with the Nintendo Wii (two Wii controllers were placed at the knee and ankle) + a motion analysis software	Standard physiotherapy alone	1 session/day (10 min or until fatigue of the participant) after surgery until discharge (average 3.2 sessions)	IKDC; Modified Cincinnati Rating System; Tegner Lysholm Knee Score	Pre-surgery baseline, before discharge, 4 weeks after surgery	No statistically significant differences were observed between groups for IKDC scores, the Cincinnati Rating scores, and the Tegner Lysholm Knee Score at 4 weeks.	4/10
Fung et al. 2012 (Canada)	Total: 50 IG: 27 CG: 23	68.1	42.0%	NR	Physiotherapy + exercise training with a Nintendo Wii Fit balance board	Physiotherapy + lower extremity exercise includes balance, posture, weight lifting and strengthening)	1 session (15 min each) in total	NPRS; ROM; 2-min walk test; Lower Extremity Functional Scale; Activity-specific Balance Confidence Scale; length of rehabilitation; satisfaction	Post-surgery baseline, at discharge (~ 50 days after surgery)	No significant differences were observed between groups for pain, knee ROM, walking speed, timed standing tasks, Lower Extremity Functional Scale, Activity-specific Balance Confidence Scale or patient satisfaction with therapy services between the groups.	5/10
Jin et al. 2018 (China)	Total: 66 IG: 33 CG:33	66.5 ± 3.5	57.6%	Knee OA	Conventional rehabilitation + rowing exercises with a VR	Conventional rehabilitation including quadriceps muscle strengthening + ROM exercises + psychological intervention + pain management education	3 sessions (30 min each)/day	WOMAC index; HSS score; VAS pain; ROM	Pre-surgery baseline, 1, 3, 6 months (WOMAC, HSS); Post-surgery baseline, 1, 3, 5, 7 days (VAS pain); Pre-surgery baseline, 3, 7, 14 days (ROM)	No significant between-group differences were found in preoperative WOMAC, HSS score and knee ROM (*P* > 0.05). WOMAC indexes were significantly lower and HSS scores were significantly higher in IG than in CG at 1, 3, and 6 months after TKA, respectively (P < 0.05). VAS pain was significantly lower in IG than CG at 3, 5, and 7 days after TKA (*P* < 0.05). Knee ROMs were significantly higher in IG than CG at 3, 7, and 14 days after TKA (P < 0.05).	6/10
Li et al. 2013 (China)	Total: 60 IG: 30 CG: 30	65 ± 12	68.3%	Knee OA	Robot-assisted walking training + VR + knee joint CPM training + peri-knee neuromuscular electrical stimulation + exercise	Knee joint CPM training + peri-knee neuromuscular electrical stimulation + exercise + walker-assisted in-door ambulation training	2 sessions/day (30 min each), 5 days/week for 2 weeks	6MWT; HSS score; knee kinesthesia grade; knee proprioception grade; FAC; Berg balance score; 10-m sitting-standing time	Post-surgery baseline, 1, 2 weeks, 1, 3, 6, 12 months	The HSS scores were significantly higher in IG compared with CG from 1 month and the difference reached a peak at 12 months. The Berg scores were significantly higher in IG from 1 week and the difference reached a peak at 3 months, lasting until the end of the study. The 10-m sitting–standing time was significantly higher in IG from 2 weeks which lasted until the end of the study. The 6MWT was higher in the IG from 1 week and the most significant difference appeared at 3 months, which lasted until the end of the study. The knee kinesthesia grade, knee proprioception grade, and FAC score were better in the IG but not statistically significant.	2/10
Web-based therapy											
Bini et al. 2016 (U.S.)	Total: 29 IG: 14 CG: 15	63.3	40.0%	NR	Standard rehabilitation programme + asynchronous educational video application on a mobile device	Standard in-person out-patient physical therapy	3 months no limit use	VAS pain; SF-36; VR-12 item health survey PCS, MCS; KOOS-PS; satisfaction	Pre-surgery baseline, 3 months	No statistically significant differences were found between groups in any of the clinical outcomes (VAS, KOOS, SF-36 PCS and MCS). There was no difference in the percentage of people that had improved more than the MCSI for both the VAS and VR-12. The overall utilization of hospital-based resources was 60% less in the IG compared with the CG.	6/10
Culliton et al. 2018 (Canada)	Total: 416 IG: 209 CG: 207	63	64%	Knee OA	Online e-learning tool during their preadmission clinic visit in addition to the 31-page guide	Standard patient education; a 31-page hard copy of “My Guide to Total Knee Joint Replacement”	12 months no limit use	Patient expectation, satisfaction, Knee Society Scoring System, KOOS, SF-12, Hospital Anxiety and Depression Scale; PCS; UCLA Activity Score; Social Role Participation Questionnaire	Pre-surgery baseline, 12 months	One year postoperatively, the risk that expectations of patients were not met was 21.8% in the CG and 21.4% in the IG for an adjusted risk difference of 1.3% (*P* = 0.78). The proportion of patients satisfied with their TKA at 12 months postoperative was 78.6% in the IG and 78.2% in CG. There are significant between-group differences in favour of the CG for the new Knee Society Knee Scoring System symptoms score (P = 0.04) and the functional activities score (*P* = 0.04) at 12 months. We also found that CG had less anxiety (P = 0.02) and lower scores for rumination (*P* = 0.02), magnification (P = 0.02), and helplessness (P = 0.02) than IG on the PCS.	7/10
Eisermann et al. 2004a (Germany)	Total: 149 IG: 75 CG:72	70	79.4%	NR	Exercise training with a computer-aided multimedia, real-time educational software	Self-training under supervision	3–5 sessions/week (30 min each) for 3–4 weeks	Staffelstein Score for TKR; Hospital for Special Surgery; FIM instrument; Hanover Functional Ability Questionnaire; patient acceptance	Post-surgery baseline, 6 months	The average functional capacity of IG has significantly improved from 46.4 ± 14.4 to 76.9 ± 16.8 (*P* < 0.001) at 6 months. The CG also increased from 48.3 ± 16.7 to 70.6 ± 20.6. Differences between follow-up and admission scores showed a small effect on the credit of the IG (effect size = 0.38). However, there was no statistically significant improvement for the IG (*P* = 0.153). The rating for acceptance of the system was 1.26 ± 0.81 in the IG compared with a rating of 1.28 ± 0.73 in the CG, which both indicated as “good” to “very good”. There was no statistically significant difference between groups.	3/10
Total hip replacement											
Telephone-based rehabilitation											
Hordam et al. 2010 (Demark)	Total: 161 IG: 68 CG: 93	74.9	62.4%	Hip OA	Conventional rehabilitation monitored via telephone support and counselling	Standard postoperative procedure	2 calls (5~15 min each) at week 2 and 10	SF-36 8 subscales	Post-surgery baseline, 12 weeks, 9 months	Physical function (*P* = 0.03), general health (*P* = 0.023) and mental health (*P* = 0.05) were significantly higher in IG compared with CG after 3 months, but all became non-significance at 9-month follow up.	6/10
Videoconferencing											
Vesterby et al. 2016 (Demark)	Total: 73 IG: 36 CG: 37	IG: 63 (43–80) CG: 64 (45–84)	47.2%	NR	Home education and medical records via a TV set + videoconferencing via the internet or mobile	In-patient and out-patient standard fast-track plan	2 videoconferences at day 2 and 6 after surgery. Total intervention for 90 days	TUGT; length of stay; HRQoL; Oxford hip score; VAS anxiety	Pre-surgery baseline, 3, 6, 12 months	HRQoL increased in both groups, but there were no statistically significant differences between groups (*P* = 0.4). There were also no statistically significant differences between groups for TUGT at 3 months and the Oxford Hip score at 3 months, 6 months or 12 months. Both groups had a statistically significant gain from baseline to 12-month follow-up (both *P* < 0.001). At the 12-month follow-up, the rates of complications and readmissions were similar between the groups, but the number of postoperative hospital contacts was lower in the IG. Length of stay was reduced from 2.1 days (95% CI: 2.0 to 2.3) to 1.1 days (95% CI: 0.9 to 1.4; P < 0.001) in the IG. Post-operative hospital contacts (phone calls) were lower in IG compared with CG at 12-month follow up (*P* = 0.04)	7/10
Web-based therapy											
Eisermann et al. 2004b (Germany)	Total: 149 IG: 79 CG: 70	68.6	70.3%	NR	Exercise training with computer-aided multimedia, real-time educational software	Self-training under supervision	3–5 sessions/week (30 min each) for 3–4 weeks	Staffelstein Score for THR; Harris Hip Score; FIM instrument; Hanover Functional Ability Questionnaire	Post-surgery baseline, 6 months	The average functional capacity of IG has significantly improved from 37.4 ± 16.8 to 72.7 ± 22.8 (*P* = 0.001) at 6 months. The CG increased in a very similar way from 38.3 ± 19.2 to 74.8 ± 23.0. There was no effect and no statistically significant difference in improvement between groups. Patients displayed their acceptance of the system by rating it with average values between “good” and “very good.” The average IG rating was 1.26 ± 0.59 compared with a rating of 1.21 ± 0.73 in the CG. There was no statistically significant difference between the two groups.	3/10
Wang et al. 2018 (China)	Total: 400 IG: 200 CG: 200	55.7 ± 13.8	53.1%	Hip OA (25%)	Interactive internet platform + videoconference	Routine rehabilitation + telephone follow-up by nurses	At least 1 chat/week for the 1st month after discharge; at least 1chat/fortnight within 2 to 4 months; at least 1 chat/month within 5 to 6 months.	Harris Hip Score; ADL; SF-36 Scale	Post-surgery baseline (admission), 3, 6 months after discharge	A significant between-group main effect was also found in favouring IG on the Harris hip scores (P < 0.001), ADL scores (*P* = 0.041) and SF-36 (*P* = 0.048).	5/10

Abbreviation *ADL* Activities of Daily Living, *CG* Control group, *CPM* Continuous passive motion, *FAC* Functional ambulation, *FIM* Functional Independence Measure, *HRQoL* Health-related quality of life, *HSS* Hospital for Specific Surgery, *KOOS* Knee injury and Osteoarthritis Outcome Score, *IKDC* International Knee Documentation Committee score, *IG* Intervention group, *LBP* Low back pain, *MCSI* Minimal Clinically Significant Improvement, *NPRS* Numerical pain rating scale, *NR* Not reported, *ODI* Oswestry Disability Index, *PCS* Pain Catastrophizing Scale, *ROM* Range of motion, *SF-12* 12 item Short Form Survey, *SF-36* Short Form-36, *SMS* Short messaging service, *6MWT* Six-minute walk test, *TKR* Total knee replacement, *THR* Total hip replacement, *TR* Telerehabilitation, *TUGT* Timed up and go test, *UCLA* University of California at Los Angeles, *VAS* Visual analogue scale, *VR* Virtual reality, *WOMAC* The Western Ontario and McMaster Universities Osteoarthritis Index

The average methodological quality of included studies was 5.8 (range: 2 to 8) on the PEDro scale (Table 1). A total of 7 studies (*N* = 1494, mean age = 65.8 years old) [27–33] were considered of high methodological quality (PEDro score ≥ 7). The most common methodological limitation was lack of blinding of the assessor observed in 10 of the 21 included trials (*N* = 1364); or therapist (16 trials, *N* = 1817).

### Details of included studies

#### Type of technologies

A total of 11 RCTs (*N* = 1596) investigated telerehabilitation via telephone counselling/coaching (6 trials, *N* = 1070) or video-conferencing (5 trials, *N* = 526). Nine RCTs (*N* = 1120, 69.7% of all participants, mean age = 67.6 years old) included people having post-TKR rehabilitation [27, 30–37] and 2 RCTs (*N* = 234, mean age = 69.2 years old) included people undergoing post-THR rehabilitation [29, 38]. There is one study in TKR that used an additional accelerometer and gyroscopes to track patient’s body movement as part of the videoconference system [37].

Game-based therapy using video games, VR or biofeedback technologies was investigated in 5 trials (*N* = 232, mean age = 64 years old) of post-TKR rehabilitation (Table 1) [28, 39–42]. In 2 studies, participants used the Wii balance board for weight-bearing and balance exercise training [28, 40]. In another study, participants were equipped with two Wii game consoles on their legs to perform knee flexion or extension exercises [39]. One trial developed a 3-D avatar in an automatic virtual environment while using a robot-assisted walking device that simulated a normal walking process in a partial weight support condition [41]. In another recent study, participants were asked to row a boat using interactive VR with robotic-assisted passive knee range of motion (ROM) exercises [42].

There were 5 eligible studies (*N* = 1143) using web-based therapies, including educational software and interactive online platform, for participants following TKR (*N* = 594, mean age = 65.4 years) or THR (*N* = 549, mean age = 62.2 years). Three studies provide multimedia online training platform used by therapists for 149 TKR and 149 THR participants, respectively [43]. Two studies use asynchronous educational software designed for handheld devices for 29 TKR participants [44].

#### Efficacy outcomes

##### Pain

**Fig. 2:**
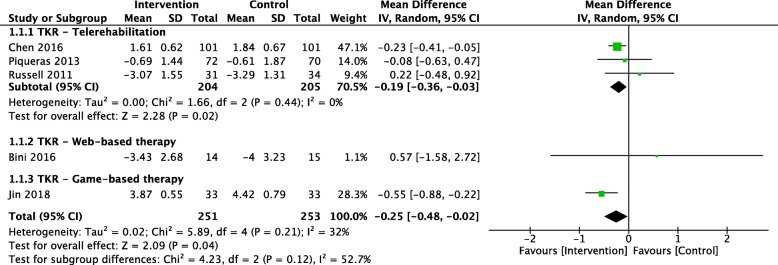
Pooled effect of trials that investigated the effects of digital rehabilitation versus usual care on the visual analogue scale for pain: scale from 0 to 10, with higher scores indicating higher pain severity. Squares represent each individual study. Diamonds represent the pooled effect. Weight (%) represents the influence of each study on the overall meta-analysis. CI, confidence interval; TKR, total knee replacement; I^2^, heterogeneity of studies

Our pooled analysis of 5 studies (*N* = 504) [27, 32, 37, 42, 44] showed that technology-assisted rehabilitation significantly improved pain measured on an 0–10-point visual analogue scale (VAS), compared to usual care, for people undergoing TKR (MD: -0.25; 95% CI: − 0.48, − 0.02) at 3 months follow up. Particularly, the subgroup analysis of telerehabilitation showed a statistically significant pain improvement (MD: -0.19; 95% CI: − 0.36, − 0.03) comparing with controls. However, both the effect sizes were too small to be of clinical significance (Fig. 2). There was no heterogeneity between the trials in telerehabilitation subgroup (*P* = 0.44; I^2^ = 0%). The quality of evidence is “moderate” due to serious risk of bias (Table 2). Due to the insufficient studies in each meta-analysis (< 10 studies), publication bias was not assessed.

**Table 2:** Summary of the quality of evidence and strength of recommendation according to Grading of Recommendations Assessment, Development and Evaluation (GRADE) criteria

Certainty assessment						№ of (events/) participants		Quality	Importance
№ of studies	Risk of bias	Inconsistency	Indirectness	Imprecision	Publication bias	Intervention	Control	Overall certainty of evidence	Importance of outcomes^a^
Pain (follow up: from 2 weeks to 3 months; assessed with: Visual Analogue Scale)									
3 RCTs [27, 32, 37]	Serious^f^	Not serious	Not serious	Not serious	None^m^	204	205	⨁⨁◯◯ Moderate	Critical
Function (follow up: range from 2 weeks to 3 months; assessed with: Timed Up and Go test)									
2 RCTs [32, 37]	Serious^f^	Serious^h^	Not serious	Serious^i^	None^m^	103	104	⨁◯◯◯ Very low	Critical
Mobility (follow up: range from 2 months to 3 months; assessed with: Six-Minute Walk Test)									
2 RCTs [31, 41]	Serious^f^	Serious^h^	Serious^g^	Very serious^i, l^	None^m^	128	130	⨁⨁◯◯ Very low	Critical
Serious adverse events^b^ (follow up: range 6 weeks to 4 months)									
3 RCTs [29–31]	Not serious	Not serious	Serious^j^	Not serious	None^m^	38/334 (11.4%)	27/333 (8.1%)^d^	⨁⨁⨁◯ Moderate	Critical
Treatment-related adverse events^c^ (follow up: range 6 weeks to 4 months)									
2 RCTs [29, 31]	Not serious	Not assessed^k^	Not serious	Not assessed^k^	None^m^	9/251 (3.1%)	8/256 (3.6%)^e^	⨁⨁⨁◯ Moderate	Critical

Abbreviations: *GRADE* Grading of recommendations assessment, development and evaluation, *RCT* Randomised controlled trial

GRADE Working Group grades of evidence

High certainty: We are very confident that the true effect lies close to that of the estimate of the effect

Moderate certainty: We are moderately confident in the effect estimate: The true effect is likely to be close to the estimate of the effect, but there is a possibility that it is substantially different

Low certainty: Our confidence in the effect estimate is limited: The true effect may be substantially different from the estimate of the effectVery low certainty: We have very little confidence in the effect estimate: The true effect is likely to be substantially different from the estimate of effect

Explanation

^a^The level of importance for patient-relevant outcome measures

^b^Serious adverse events include: hospital readmission for leg blister, manipulation under aesthesia for poor knee range of motion, prostate check and cataract surgery (Han 2015); death, hospitalization, manipulation under aesthesia, degradation of the general condition, hip fracture due to fall, gastrointestinal disorder, rheumatologic disorder, cardiac arrhythmia, thrombophlebitis, spinal surgery, inguinal hernia surgery, cystocele surgery, retinal detachment surgery, total knee arthroplasty on contralateral side (Moffet 2016)

^c^Treatment-related adverse events include: operated knee swelling and/or extreme knee pain; excess wound leakage or bleeding (Han et al., 2015)

^d^Risk difference with intervention: 33 more per 1000 (from 9 fewer to 100 more)

^e^Risk difference with intervention: 6 more per 1000 (from 17 fewer to 67 more)

^f^More than 25% of participants from studies with low methodological quality (Physiotherapy Evidence Database score < 7 points)

^g^Different technologies were analysed together (Moffet 2016 – telerehabilitation; Li 2014 – game-based therapy)

^h^I^2^ > 50%; substantial heterogeneity

^i^Small sample size: < 400 participants in the pooling.

^j^A mixed population of hip and knee replacement: 10% of patients have total hip replacement (Vesterby 2016 – hip replacement-only study)

^k^Zero events were reported in one of the trials.

^l^95% CI overlaps no effects (i.e. fails to exclude important benefit or important harm)

^m^The possibility of publication bias is not excluded but it was not considered as sufficient to downgrade the quality of evidence

##### Function

**Time up and Go test (TUGT)**

**Fig. 3:**
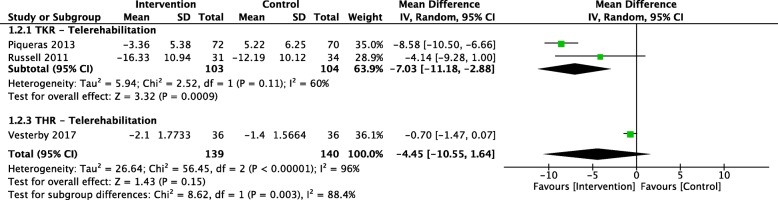
Pooled effect of trials that investigated the effects of digital rehabilitation versus usual care on timed up and go test: assessed in second, with a higher number indicating worse functional ability. Squares represent each individual study. Diamonds represent the pooled effect. Weight (%) represents the influence of each study on the overall meta-analysis. CI, confidence interval; TKR, total knee replacement; THR, total hip replacement; I^2^, heterogeneity of studies

Our analyses pooling 2 studies (*N* = 207) [32, 37] showed that telerehabilitation significantly improved function, assessed via the TUGT (measured by second; less time spend indicates better function) [45] over a short term (2 weeks to 3 months), compared with usual rehabilitation for people following TKR (MD: -7.03; 95% CI: − 11.18, − 2.88). There was a substantial heterogeneity (*P* = 0.11; I^2^ = 60%). No difference was observed for those undergoing THR (MD: -0.70; 95% CI: − 1.47, 0.07) (Fig. 3). The quality of evidence was considered as “very low” because of the serious risk of bias, inconsistency and imprecision (Table 2).

**6 minute walking test (6MWT)**

**Fig. 4:**
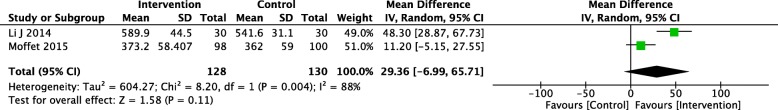
Pooled effect of trials that investigated the effects of digital rehabilitation versus usual care on six-minute walk test: assessed in metre, with a higher number indicating better mobility. Squares represent each individual study. Diamonds represent the pooled effect. Weight (%) represents the influence of each study on the overall meta-analysis. CI, confidence interval; TKR, total knee replacement; I^2^, heterogeneity of studies

There were two RCTs (*N* = 258) [31, 41] assessed mobility via 6MWT (measured by metre; longer distance indicates better mobility) [46] showing technology-assisted rehabilitation is not significantly superior to usual care in people who underwent TKR (MD: 29.36; 95% CI: − 6.99, 65.71) at the short-term (2 to 3 months) (Fig. 4). A high heterogeneity was detected (*P* < 0.01; I^2^ = 88%). The quality of evidence was downgraded to “very low” due to serious risk of bias, inconsistent results between 2 studies and indirectness of interventions (i.e. tele-rehabilitation and robotic-assisted VR were analysed together) (Table 2).

**Western Ontario and McMaster Universities Osteoarthritis Index (WOMAC)**

Four trials reported change in WOMAC on a 5-point Likert scale (standardised to 0–4 for each subscale) [47] (*N* = 746) [27, 30, 32, 33], 3 trials reported pain (*N* = 560) [30, 32, 33] and stiffness (*N* = 371) subscales [27, 32, 33]. There is low-to-moderate quality, downgraded for serious inconsistency and imprecision (data not shown), that telerehabilitation is not superior to usual care in improving WOMAC pain (MD: -0.09; 95% CI: − 0.22, 0.04; I^2^ = 15%; moderate evidence), function (MD: -0.05; 95% CI: − 0.16, 0.06; I^2^ = 34%; moderate evidence) or stiffness (MD: -0.07; 95% CI: − 0.32, 0.17; I^2^ = 67%; very low evidence) at the 3 months follow-up (Additional file 2: Fig. S1, S2 and S3).

#### Secondary outcomes

##### Quality of life

Six trials (TKR: *N* = 520; THR: *N* = 161) investigated the effect of telerehabilitation on quality of life (Table 1) [27, 33, 34, 36, 38, 44]. Meta-analysis was not feasible due to differences in completeness of reported data and inconsistent measurements. Two trials in people who underwent rehabilitation after TKR reported that telerehabilitation showed significant improvements on short form (SF)-36 mental component score (*P* < 0.01) [27] and physical function subscale (*P* = 0.031) [33], respectively. One study of THR showed physical function (P = 0.03), general health (*P* = 0.023) and mental health (*P* = 0.05) subscales of SF-36 were all significantly higher in the telerehabilitation group compared with the control group after 3 months, but all became non-significance at 9-month follow-up [38].

##### Adherence and user experience

Three RCTs of 472 people undergoing TKR investigated their compliance through an exercise diary [27, 31, 32]. One study showed the average time of daily home exercise in the telerehabilitation group (54.12 ± 5.71 mins) was significantly higher than the control group (48.95 ± 7.21 mins) [27]. Two studies showed no between-group differences in the number of exercise sessions finished daily [31, 32].

Four trials (*N* = 757) reported user experience and showed similar levels of satisfaction with both the intervention and the control [40, 43, 44, 48]. One trial of an educational software demonstrated positive user experiences, such as good clarity of instruction, ease of taking or sharing a video and ease of seeing their progress [44]. Another study of training software also received positive feedback from participants and therapists [43]. When participants were asked what they liked most about the application, no travelling to the hospital was cited by 57% and ease of access by 21% [44].

##### Safety

Moderate quality evidence from 3 RCTs (*N* = 667) showed the total number of serious adverse events (SAEs) were higher in the intervention group comparing to usual care (38 vs. 27) [29–31] (Table 2). However, there were no SAEs related to the intervention, while 2 events in the usual care group: one fell and one had wound bleeding during the first knee flexion exercise [31]. Of all the patients who had hospital admissions related knee issues, one in the usual care group had a leg blister below the TKR site, 3 in the usual care and 4 in the telerehabilitation group received manipulation under anaesthesia [30, 31]; one participant in the telerehabilitation group had thrombophlebitis [31]. One THR patient in the intervention group had a fever [29].

## Discussion

Our review found that moderate-quality of evidence showed technology-assisted rehabilitation, in particular, telerehabilitation, had a statistically significant improvement in pain; and low-quality of evidence for the improvement in functional mobility in people undergoing TKR. The effects were however small and of arguable clinical significance. For THR, there is very limited low-quality evidence shows no significant effects. Pre-planned sub-group meta-analyses on study design (i.e. technology-based rehabilitation alone or in addition to usual care) were not performed due to insufficient studies. Most of the trials only had short-term follow-ups, therefore, the long-term effectiveness of technology-assisted rehabilitation was not ascertained.

Compared to previous studies in the field, our review has identified more than twice the number of the trials and most of the new studies added in our meta-analyses had higher methodological quality. For instance, the most recent systematic review only included 8 RCTs of post-TKR rehabilitation and 3 RCTs of post-THR rehabilitation and only provided a qualitative evaluation of those studies [15]. It concluded that the evidence was strong based on a PEDro score ≥5, which seems to be overestimated [49].

From the few studies that investigated user experience, there is a trend towards a positive impact of telerehabilitation, particularly, adherence to physical activities and compliance to rehabilitation programs [27, 31, 32]. Although the majority of the study population were older adults, their use of technologies, such as smartphone was quite high (59–49%) [50]. Similarly, in older adults with no prior experience with game consoles, most of them were highly motivated and expressed enjoyment in using the Wii Fit [39] and 86% of them were willing to continue the game therapy at home [40]. Some barriers were also demonstrated, such as poor internet connection at the participant’s home, delayed technology installation [32] and poor visual quality of the video-conference [32]. Additionally, older people may experience technological adoption barriers, such as concerns about the cost and battery life of the devices, as well as lack of familiarity with the technology [51]. These highlighted the need for cost-effective and power-efficient devices, elderly user-friendly design, sufficient training and ongoing customer support.

Importantly, the innovative devices or digital technologies should not be viewed as a distinct modality of care, but rather used as an aid/adjunct to bridge gaps or accelerate efficiency in existing healthcare delivery systems [52]. A study showed that telerehabilitation in addition to usual care was more favourable than usual care alone, whilst treatment delivered solely via telerehabilitation was equivalent to face-to-face intervention for functional improvement in people with MSK conditions [16]. In addition, validity studies reported a good agreement between face-to-face and telehealth assessment of MSK disorders of the knee (exact agreement of primary pathoanatomical diagnoses was 67%) [53]. Given the fact that technology could improve the healthcare accessibility and treatment adherence, despite its clinical effectiveness was similar comparing to conventional intervention, it still has a very promising role in circumstances when access and adherence are challenging.

Apart from some practical issues of licensure, there are potential challenges when implementing digital technologies in clinical practice. Firstly, the safety of the technology-assisted rehabilitation needs to be better understood. In our review, only a handful of studies reported AEs, although they all showed no increased harm. For game-based therapy, trials in the current review did not report any AEs, but it is reported that dynamic movements followed by different games can increase falls risks or other MSK injuries [54]. Safeguards should be taken pre-emptively when emergencies need to be solved virtually [55]. Healthcare providers embarking on careers in innovative technologies should be aware of current legal regulations to minimise risk [55]. Cost can also be a barrier when certain technology was first developed, thus, high-quality cost-effectiveness analyses are needed to demonstrate the long-term economic benefits.

There are several limitations to our review. Many studies did not perform a priori sample size calculations, which can increase the risk of underpowered (false-negative) results. Secondly, the trials used varied outcome measures which limited the pooling of results. Consensus on a set of suitable outcome measures needs to be reached for future trials. Furthermore, there is insufficient long-term follow up for ensuring the prolonged effects or safety. Lastly, a common risk of bias of the studies is a lack of blinding. As blinding of participants and therapists is not possible for most pragmatic trials, including those of technology-based rehabilitation interventions, future research should pay attention to the methodological aspects to minimise the biases.

## Conclusion

There is moderate- to low-quality of evidence that current technology-enabled rehabilitation, in particular, telerehabilitation, showed most improvements in pain and function for people following TKR, comparing to usual rehabilitation. However, the effect size was too small to be clinically significant. Further high-quality studies are needed to demonstrate the long-term efficacy and safety of innovative health technologies, especially for post-THR rehabilitation.

## Supplementary information

**Additional file 1: Appendix 1.** Search strategies

**Additional file 2: Figure S1.** Pooled effect of trials that investigated the effects of digital rehabilitation versus usual care on the Western Ontario and McMaster Universities Osteoarthritis Index function scores (5-point Likert scale). Squares represent each individual study. Diamonds represent the pooled effect. Weight (%) represents the influence of each study on the overall meta-analysis. CI, confidence interval; I^2^, heterogeneity of studies. **Figure S2.** Pooled effect of trials that investigated the effects of digital rehabilitation versus usual care on the Western Ontario and McMaster Universities Osteoarthritis Index pain scores (5-point Likert scale). Squares represent each individual study. Diamonds represent the pooled effect. Weight (%) represents the influence of each study on the overall meta-analysis. CI, confidence interval; I^2^, heterogeneity of studies. **Figure S3.** Pooled effect of trials that investigated the effects of digital rehabilitation versus usual care on the Western Ontario and McMaster Universities Osteoarthritis Index stiffness scores (5-point Likert scale). Squares represent each individual study. Diamonds represent the pooled effect. Weight (%) represents the influence of each study on the overall meta-analysis. CI, confidence interval; I^2^, heterogeneity of studies.

## Data Availability

All data generated or analysed during this study are included in this published article [and its supplementary information files].

## Abbreviations

6MWT: 6 Minute Walking Test
GRADE: Grading of Recommendations, Assessment, Development and Evaluation
ICCs: Intraclass correlation coefficient
MD: Mean difference
MSK: Musculoskeletal
NHMRC: National Health and Medical Research Council
ORs: Odds ratios
PEDro: Physiotherapy Evidence Database
PRISMA: Preferred Reporting Items for Systematic Reviews and Meta-Analysis
RCTs: Randomised controlled trials
RRs: Risk ratios
SAEs: Serious adverse events
SDs: Standard deviations
SEs: Standard errors
SF: Short form
SMD: Standardised MDs
THR: Total hip replacement
TKR: Total knee replacement
TUGT: Time up and Go Test
VR: Virtual reality
WOMAC: Western Ontario and McMaster Universities Osteoarthritis Index
