# Supplementary material for: Technology-assisted rehabilitation following total knee or hip replacement for people with osteoarthritis: a systematic review and meta-analysis
Source: BMC Musculoskelet Disord. 2019 Nov 3;20:506. doi: 10.1186/s12891-019-2900-x (PMC6825714; doi:10.1186/s12891-019-2900-x)
Supplement: Supplementary file 1 — Additional file 1: Appendix 1. Search strategies [file 12891_2019_2900_MOESM1_ESM.docx]

**Additional file 1: Appendix 1. Search strategies**

MEDLINE, EMBASE and Cochrane Central Register of Controlled Trials via OvidSP

(/indicates a MEDLINE index term)

|  | Search terms |
| --- | --- |
|  | Arthroplasty, Replacement, Knee/ |
|  | Knee Prosthesis/ |
|  | Arthroplasty, Replacement, Hip/ |
|  | Hip Prosthesis/ |
|  | ((hip* or knee*) adj3 (replac* or arthroplast* or implant* or endoprosthe* or prosthe*)).mp. |
|  | Total Disc Replacement/ |
|  | Intervertebral Disc Displacement/ |
|  | exp Arthrodesis/ |
|  | exp Decompression, Surgical/ |
|  | Foraminotomy/ |
|  | Laminectomy/ |
|  | Laminoplasty/ |
|  | Radiculopathy/ |
|  | (((((spin* or lumb* or low* back or back) and (pain or ache)) or backpain or backache or spondylo* or (hernia* adj3 dis#) or sciatic* or vertebral canal or (lumbar adj3 radiculopathy) or intervertebral dis#) and (surg* or operat* or orthopaedic* or procedure* or displacement or arthrodes#s or lamin* or dis#ectomy or forami* or facetectomy or osteotom* or nucleotomy or microdiscectomy or nucleoplasty or decompress* or spondylo* or (bone adj3 graft) or (spinal adj3 fixation) or (pedicle adj3 fusion)))).mp. |
|  | 1 – 13 or |
|  | (((after or post) and (care or surg* or operat*)) or ((exercis* or fitness or physical or neuromuscular or functional) adj3 (therapy or educat* or training)) or aftercare or postsurg* or postoperat* or rehab* or home care or homecare).mp. |
|  | Postoperative Care/ |
|  | Rehabilitation Research/ |
|  | exp Rehabilitation/ |
|  | exp Aftercare/ |
|  | exp Exercise Therapy/ |
|  | exp Physical Fitness/ |
|  | exp Home Care Services/ |
|  | 15 – 22 or |
|  | Telerehabilitation/ |
|  | Telemedicine/ |
|  | Biosensing Techniques/ |
|  | Virtual Reality Exposure Therapy/ |
|  | exp Telephone/ |
|  | exp Telemetry/ |
|  | exp Videoconferencing |
|  | exp Software/ |
|  | exp Internet/ |
|  | exp Accelerometry/ |
|  | exp Monitoring, Ambulatory/ |
|  | exp Biofeedback, Psychology/ |
|  | (((tele* or mobile or phone or online or internet or digital) and (rehab* or care or medicine or health)) or telehealth or telemedicine or telerehab* or mhealth or messag* or smart* or (social adj3 (device or platform)) or software or computer or website or web based or ((digital or handheld or electronic*) adj3 (device or tablet)) or wearable* or ((motion or movement or activity or mobility) adj3 (sens* or monitor* or track*)) or biofeedback or accelerometer* or pedometer* or gyroscope or actimetry or ((virtual or video or electronic*) and (gam* or learn* or educat* or conferenc*)) or virtual or Nintendo or playstation).mp. |
|  | 24 – 36 or |
|  | Control Groups/ |
|  | Single-Blind Method/ |
|  | Double-Blind Method/ |
|  | Random Allocation/ |
|  | Placebos/ |
|  | exp Controlled Clinical Trial/ |
|  | exp Clinical Trials as Topic/ |
|  | (random* or placebo* or controlled clinical trial or groups or randomly or ((single or double or triple) adj3 blind*) or (random* adj3 (trial or study))).ti,ab. |
|  | (randomized controlled trial or controlled clinical trial).pt. |
|  | 36 – 44 or |
|  | 14 and 23 and 37 and 47 |
|  | limit 48 to human |

EMBASE

|  | Search terms |
| --- | --- |
|  | Arthroplasty, Replacement, Knee/ |
|  | Knee Prosthesis/ |
|  | Arthroplasty, Replacement, Hip/ |
|  | Hip Prosthesis/ |
|  | ((hip* or knee*) adj3 (replac* or arthroplast* or implant* or endoprosthe* or prosthe*)).mp. |
|  | Total Disc Replacement/ |
|  | Intervertebral Disc Displacement/ |
|  | exp Arthrodesis/ |
|  | exp Decompression, Surgical/ |
|  | Foraminotomy/ |
|  | Laminectomy/ |
|  | Laminoplasty/ |
|  | (((((spin* or lumb* or low* back or back) and (pain or ache)) or backpain or backache or spondylo* or (hernia* adj3 dis#) or sciatic* or vertebral canal or (lumbar adj3 radiculopathy) or intervertebral dis#) and (surg* or operat* or orthopaedic* or procedure* or displacement or arthrodes#s or lamin* or dis#ectomy or forami* or facetectomy or osteotom* or nucleotomy or microdiscectomy or nucleoplasty or decompress* or spondylo* or (bone adj3 graft) or (spinal adj3 fixation) or (pedicle adj3 fusion)))).mp. |
|  | 1 – 13 or |
|  | (((after or post) and (care or surg* or operat*)) or ((exercis* or fitness or physical or neuromuscular or functional) adj3 (therapy or educat* or training)) or aftercare or postsurg* or postoperat* or rehab* or home care or homecare).mp. |
|  | Postoperative Care/ |
|  | Rehabilitation Research/ |
|  | exp Rehabilitation/ |
|  | exp Aftercare/ |
|  | exp Exercise Therapy/ |
|  | exp Physical Fitness/ |
|  | exp Home Care Services/ |
|  | 15 – 22 or |
|  | Telerehabilitation/ |
|  | Telemedicine/ |
|  | Biosensing Techniques/ |
|  | Virtual Reality Exposure Therapy/ |
|  | exp Telephone/ |
|  | exp Telemetry/ |
|  | exp Videoconferencing |
|  | exp Software/ |
|  | exp Internet/ |
|  | exp Accelerometry/ |
|  | exp Monitoring, Ambulatory/ |
|  | exp Biofeedback, Psychology/ |
|  | (((tele* or mobile or phone or online or internet or digital) and (rehab* or care or medicine or health)) or telehealth or telemedicine or telerehab* or mhealth or messag* or smart* or (social adj3 (device or platform)) or software or computer or website or web based or ((digital or handheld or electronic*) adj3 (device or tablet)) or wearable* or ((motion or movement or activity or mobility) adj3 (sens* or monitor* or track*)) or biofeedback or accelerometer* or pedometer* or gyroscope or actimetry or ((virtual or video or electronic*) and (gam* or learn* or educat* or conferenc*)) or virtual or Nintendo or playstation).mp. |
|  | 24 – 36 or |
|  | Control Groups/ |
|  | Single-Blind Method/ |
|  | Double-Blind Method/ |
|  | Random Allocation/ |
|  | Placebos/ |
|  | exp Controlled Clinical Trial/ |
|  | exp Clinical Trials as Topic/ |
|  | (random* or placebo* or controlled clinical trial or groups or randomly or ((single or double or triple) adj3 blind*) or (random* adj3 (trial or study))).ti,ab. |
|  | (randomized controlled trial or controlled clinical trial).pt. |
|  | 36 – 44 or |
|  | 14 and 23 and 37 and 47 |
|  | exp cohort analysis/ |
|  | exp prospective study/ |
|  | exp longitudinal study/ |
|  | exp meta analysis/ |
|  | exp Systematic Review/ |
|  | exp case control study/ |
|  | cohort*.tw. or or (case* and control*).tw. or exp case study/ or (case* and series).tw. or (book or conference paper or editorial or letter or review).pt. or (literature adj3 review*).ti,ab. or or (systematic* adj2 (review* or overview)).ti,ab. or (guideline*).ti or exp Practice Guideline/ |
|  | 9 not 10 |
|  | limit 48 to human |

CINAHL and SPORTDiscus via EBSCO

|  | Search terms |
| --- | --- |
|  | (MH "Arthroplasty, Replacement, Knee+") OR (MH "Arthroplasty, Knee, Unicompartmental") |
|  | (MH "Arthroplasty, Replacement, Hip") |
|  | (TX hip* or TX knee*) N3 (TX replac* or TX arthroplast* or TX implant* or TX endoprosthe* or TX prosthe*) |
|  | (MH "Intervertebral Disk Displacement") |
|  | (MH "Arthrodesis+") |
|  | (MH "Decompression, Surgical+") |
|  | (MH "Laminectomy") |
|  | (MH "Laminoplasty") |
|  | ((((TX spin* or TX lumb* or TX low* back or TX back) and (TX pain or TX ache)) or TX backpain or TX backache or TX spondylo* or (TX hernia* N3 dis#) or TX sciatic* or TX vertebral canal or (TX lumbar N3 TX radiculopathy) or TX intervertebral dis#) and (TX surg* or TX operat* or TX orthopaedic* or TX procedure* or TX displacement or TX arthrodes#s or TX lamin* or TX dis#ectomy or TX forami* or TX facetectomy or TX osteotom* or TX nucleotomy or TX microdiscectomy or TX nucleoplasty or TX decompress* or TX spondylo* or (TX bone N3 TX graft) or (TX spinal N3 TX fixation) or (TX pedicle N3 TX fusion))) |
|  | 1 – 9 or |
|  | (((TX after or TX post) and (TX care or TX surg* or TX operat*)) or ((TX exercis* or TX fitness or TX physical or TX neuromuscular or TX functional) N3 (TX therapy or TX educat* or TX training)) or TX aftercare or TX postsurg* or TX postoperat* or TX rehab* or TX home care or TX homecare) |
|  | (MH "Postoperative Care") |
|  | (MH "Rehabilitation+") |
|  | (MH "Aftercare") |
|  | (MH "Therapeutic Exercise+") |
|  | (MH "Physical Fitness+") |
|  | (MH "Exercise+") |
|  | 11 – 17 or |
|  | (MH "Communications Media+") |
|  | (MH "Telehealth+") |
|  | (MH "Biosensing Techniques+") |
|  | (MH "Video Games+") |
|  | (MH "Computer Simulation+") |
|  | (MH "Telemetry") |
|  | (MH "Software+") |
|  | (MH "Interactive Voice Response Systems") |
|  | (MH "Internet+") |
|  | (MH "Accelerometry+") |
|  | (MH "Electrocardiography, Ambulatory") |
|  | (MH "Biofeedback") |
|  | (((TX tele* or TX mobile or TX phone or TX online or TX internet or TX digital) and (TX rehab* or TX care or TX medicine or TX health)) or TX telehealth or TX telemedicine or TX telerehab* or TX mhealth or TX messag* or TX smart* or (TX social N3 (TX device or TX platform)) or TX software or TX computer or TX website or TX web based or ((TX digital or TX handheld or TX electronic*) N3 (TX device or TX tablet)) or TX wearable* or ((TX motion or TX movement or TX activity or TX mobility) N3 (TX sens* or TX monitor* or TX track*)) or TX biofeedback or TX accelerometer* or TX pedometer* or TX gyroscope or TX actimetry or ((TX virtual or TX video or TX electronic*) and (TX gam* or TX learn* or TX educat* or TX conferenc*)) or TX virtual or TX Nintendo or TX playstation) |
|  | 19 – 31 or |
|  | (PT randomized controlled trial OR PT controlled clinical trial) |
|  | (MH "Clinical Trials+") |
|  | (MH "Control Group") |
|  | (MH "Random Sample+") |
|  | (MH "Placebos") |
|  | (AB controlled clinical trial or AB placebo* or AB groups or AB randomly or (AB random* N3 (trial or study)) or ((AB single or AB double or AB triple) N3 blind*)) |
|  | (PT randomized controlled trial or PT controlled clinical trial) |
|  | 33 – 39 or |
|  | 10 and 18 and 32 and 40 |
|  | limit 41 to human |
|  | (MH "Case Control Studies+") |
|  | (MH "Matched-Pair Analysis") |
|  | (MH “Cohort Studies”) |
|  | (MH “Systematic Review”) |
|  | (MH “Meta Analysis”) |
|  | ((TI (case or cases) n5 TI (control or controls)) OR (AB (case or cases) n5 AB (control or controls)) OR (TI (case or cases) n3 TI (matched)) OR (AB (case or cases) n3 AB (matched)) OR TI (control group*) cohort [TIAB] OR longitudinal [TIAB] OR prospective [TIAB] OR retrospective [TIAB] OR (TI (meta-analy* OR metaanaly*)) OR (AB (meta-analy* OR metaanaly*)) |
|  | 43 – 48 or |
|  | 42 not 49 |
