# Supplementary material for: Technology-assisted rehabilitation following total knee or hip replacement for people with osteoarthritis: a systematic review and meta-analysis
Source: BMC Musculoskelet Disord. 2019 Nov 3;20:506. doi: 10.1186/s12891-019-2900-x (PMC6825714; doi:10.1186/s12891-019-2900-x)
Supplement: Supplementary file 2 — Additional file 2: Figure S1. Pooled effect of trials that investigated the effects of digital rehabilitation versus usual care on the Western Ontario and McMaster Universities Osteoarthritis Index function scores (5-point Likert scale). Squares represent each individual study. Diamonds represent the pooled effect. Weight (%) represents the influence of each study on the overall meta-analysis. CI, confidence interval; I2, heterogeneity of studies. Figure S2. Pooled effect of trials that investigated the effects of digital rehabilitation versus usual care on the Western Ontario and McMaster Universities Osteoarthritis Index pain scores (5-point Likert scale). Squares represent each individual study. Diamonds represent the pooled effect. Weight (%) represents the influence of each study on the overall meta-analysis. CI, confidence interval; I2, heterogeneity of studies. Figure S3. Pooled effect of trials that investigated the effects of digital rehabilitation versus usual care on the Western Ontario and McMaster Universities Osteoarthritis Index stiffness scores (5-point Likert scale). Squares represent each individual study. Diamonds represent the pooled effect. Weight (%) represents the influence of each study on the overall meta-analysis. CI, confidence interval; I2, heterogeneity of studies. [file 12891_2019_2900_MOESM2_ESM.docx]

**Additional file 2: Figures legend**

Figure S1. Pooled effect of trials that investigated the effects of digital rehabilitation versus usual care on the Western Ontario and McMaster Universities Osteoarthritis Index function scores (5-point Likert scale). Squares represent each individual study. Diamonds represent the pooled effect. Weight (%) represents the influence of each study on the overall meta-analysis. CI, confidence interval; I^2^, heterogeneity of studies.

Figure S2. Pooled effect of trials that investigated the effects of digital rehabilitation versus usual care on the Western Ontario and McMaster Universities Osteoarthritis Index pain scores (5-point Likert scale). Squares represent each individual study. Diamonds represent the pooled effect. Weight (%) represents the influence of each study on the overall meta-analysis. CI, confidence interval; I^2^, heterogeneity of studies.

Figure S3. Pooled effect of trials that investigated the effects of digital rehabilitation versus usual care on the Western Ontario and McMaster Universities Osteoarthritis Index stiffness scores (5-point Likert scale). Squares represent each individual study. Diamonds represent the pooled effect. Weight (%) represents the influence of each study on the overall meta-analysis. CI, confidence interval; I^2^, heterogeneity of studies.

Figure S1

Figure S2

Figure S3
